# Ultrasound-Induced Blood-Brain-Barrier Opening Enhances Anticancer Efficacy in the Treatment of Glioblastoma: Current Status and Future Prospects

**DOI:** 10.1155/2019/2345203

**Published:** 2019-11-03

**Authors:** Zhiting Deng, Zonghai Sheng, Fei Yan

**Affiliations:** ^1^Paul C. Lauterbur Research Center for Biomedical Imaging, Institute of Biomedical and Health Engineering, Shenzhen Institutes of Advanced Technology, Chinese Academy of Sciences, Shenzhen 518055, China; ^2^Shenzhen Key Laboratory of Ultrasound Imaging and Therapy, Shenzhen 518055, China

## Abstract

Glioblastoma multiforme (GBM) diffusely infiltrates normal brain tissue. The presence of the blood-brain barrier (BBB) poses difficulties for targeted delivery of currently available antitumor drugs. Novel brain drug delivery strategies are far from satisfactory for glioma treatment. Recently, focused ultrasound (FUS) combined with microbubbles presents a transient, reversible, and noninvasive approach for local induction of BBB opening. This strategy demonstrated its potential to increase local concentrations of both diagnostic and therapeutic agents in glioma therapy. Current status and related physic mechanisms of this drug delivery technique are discussed in this review. Delivery efficiency enhancement in many preclinical glioma models was obtained by FUS-BBB opening combined with various nanoparticles. And, the clinical translational status of FUS-BBB will be discussed.

## 1. Introduction

Glioblastoma multiforme (GBM), characterized by high recurrence and poor prognosis, is the most malignant primary brain tumor in adults [1, 2]. The prognosis of patients with gliomas still remains very poor after tremendous efforts in both basic and clinical research. Patients were treated by adjuvant chemotherapy and radiation therapy after aggressive surgery. However, the 5-year survival rate is less than 5% and with approximately 12–14 months of median overall survival durations [3].

The blood-brain barrier (BBB) is a highly specialized structure in brain [4]. The BBB is formed by brain microvascular endothelial cells. Endothelial cells (EC) were sealed by tight junctions (TJs), basement membrane of pericyte, and end-feet of astrocyte. It acts as a selective physical barrier for maintaining the homeostasis of the brain by regulating immune cell transport, passive diffusion of chemicals, and entry of xenobiotics [5, 6]. In physiology, the BBB selectively allows only certain substances to pass between the brain tissue and the blood vessels. Therefore, the BBB protects the brain from possible toxic elements and unfortunately also severely prevents potential antitumor drugs into glioma. In this case, it is very necessary and important to disrupt the BBB, `allowing the diffusion or delivery of therapeutic or diagnostic agents into the brain [7–9].

Various approaches were developed to overcome the BBB problem in glioma treatment. Gliadel wafer, biodegradable 1,3-bis (2-chloroethyl)-1-nitrosourea (BCNU) containing wafer, was approved by FDA in 1995 for glioma treatment [10]. After glioma resection, they were implanted on the surface of the surgical resection cavity for local release of BCNU. It could bypass BBB because it involves local surgical treatment. Convection-enhanced delivery (CED) directly delivers high concentrations of drug within and around brain tumors by surgical placement of catheters into the brain. It also bypasses the blood-brain barrier and limits systemic exposure of chemotherapeutics. CED of drugs in glioma has shown promise in animal studies and clinical trials [11, 12]. However, this method also needs invasive surgical exposure of the brain.

Ultrasound refers to mechanical waves with frequencies greater than 20 kHz, the audible limit of human hearing. Ultrasound is best known as a real-time, noninvasive medical imaging method using sound waves. Focused ultrasound (FUS) enables producing precisely focused acoustic energy within a small volume noninvasively [13]. Focused ultrasound (FUS) combined with microbubbles can locally disrupt the BBB.

This procedure is usually noninvasive and reversible within several hours, after systemically injecting the therapeutic agents, thus providing great potential for therapeutic agents to extravasate into targeted glioma area [14].

### 1.1. Mechanisms of BBB Disruption with Focused Ultrasound

**Figure 1:**
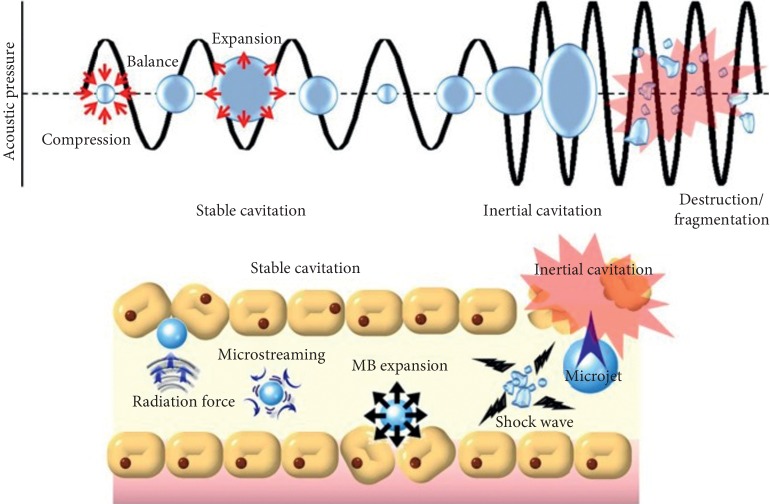
Physical mechanisms underlying FUS-BBB opening (reproduced from [7], an open-access journal printed by the Ivyspring International Publisher, free to use).

Focused ultrasound- (FUS-) induced BBB disruption is facilitated by microbubbles. Microbubbles expand and contract upon sonications by FUS, producing cavitation effects. In general, there are two kinds of cavitations: stable and inertial cavitations, as shown in Figure 1. Stable cavitation is induced by relatively lower amplitude of FUS, referring to repetitive contractions and expansions of microbubbles [15]. Microstreamings are formed around oscillating microbubbles, and cells near around would experience shear stress, eventually producing pore formation on the cell membrane. Inertial cavitation occurs when acoustic pressure is amplified, and then microbubbles would be destructed or collapsed [7]. When shockwaves and microjets are generated, tight junctions would be temporally disassembled, vascular permeability would increase, and thus drug transportation would enhance eventually [16, 17].

To induce these biological effects, utilization of microbubbles is important. Microbubbles could significantly reduce the US power level by two orders of magnitude at least, when compared with US without microbubbles [18]. Microbubbles, microsized microspheres, are filled with hydrophobic gas like perfluorocarbons or sulfur hexafluoride. Microbubbles are usually manufactured by lipid, denatured protein (albumin), surfactant, or polymer. Three kinds of commercially available microbubbles: Optison, Definity [19], and SonoVue were proved to open BBB with FUS successfully [20, 21].

Besides microbubbles, different ultrasound parameters also showed various effects on BBB disruption. Choi et al. showed that even very short pulses of ultrasound waves could open the blood-brain barrier (BBB). In their study, a 3.5-cycle (2.3-*μ*s) pulse was emitted by FUS. Microbubbles and fluorescent-labeled dextrans were administered i.v and the confocal microscopy results revealed the diffusion of dextrans with different molecular weight after BBB disruption [22]. Chen and Konofagou found that during FUS-BBB opening, compared with other parameters, higher acoustic pressure was responsible for dextrans with larger molecular weight to pass BBB [23]. Their data indicated that different molecular sizes which were allowed to penetrate through BBB need different acoustic pressures. It also means that it is possible to select appropriate acoustic pressure to deliver the drugs according to their molecular sizes. Generally, various acoustic parameters, such as pulse repetition frequency, exposure time, [24] pressure amplitude, [24], and pulse length [25], were fully tested in the previous research. And different kinds of microbubbles [21, 26] and dosage of microbubbles [25] were also contributed to different BBB opening effects. For example, Marquet et al. utilized FUS at 500 kHz frequency to achieve BBB opening in nonhuman primates. The acoustic parameters were listed as follows: pressure amplitude at 0.3 MPa and 0.45 MPa, pulse length of 10 ms, PRF at 2 Hz, and total exposure time of 120 s. Two kinds microbubbles—Definity and customized microbubbles—were used in this study [27].

Most studies have shown that BBB is restored in a few hours after sonication. However, the duration of BBB opening could stay open for days and even can be permanently damaged when parameters are suboptimal. A linear proportionate was found between the sonication duration and the opening size of BBB [28]. The magnitude of FUS-BBB opening differs greatly between studies because of various sonication parameters. Over the past years, many safety studies of FUS-BBB opening have been thoroughly investigated including histopathological changes, neural function, physiological effects, biochemical assays [29], and behavior tasks [30]. For a safe and successful clinical translation of FUS-BBB opening, real-time feedback control of acoustic parameters has been developed recent years [31, 32]. The passive cavitation detector (PCD) was used by Maimbourg et al. to quantify acoustic signal of microbubbles when sonicated by focused ultrasound [33]. The feedback control algorithm for real-time monitoring of microbubbles' acoustic emissions under FUS has been evaluated by Bing et al. [21].

### 1.2. FUS-BBB Opening for Targeted Chemotherapeutics Delivery in Glioma Treatment

Many chemotherapeutics fail to cross the BBB, and thus the clinical application has been severely limited. Doxorubicin (DOX) is a widely used chemotherapeutic agent with excellent antineoplastic efficacy. However, DOX does not cross the BBB. Its insufficient accumulation in brain tissue resulted in poor efficacy in glioma treatment. Taking advantage of MRI-guided focused ultrasound to open the BBB, Treat et al. achieved enough concentrations of DOX in the brain tumors, and antitumor efficacy was also significantly enhanced [34]. Another example is 1,3-bis (2-chloroethyl)-1-nitrosourea (BCNU), a chemotherapy drug used for glioma, which showed only a relatively limited effect in the short-term survival study. By contrast, Liu et al. employed FUS-BBB for delivery of BCNU to glioma-bearing rats. It greatly increased the concentration of BCNU in normal brains (by 340%) and tumor-bearing (by 202%) brains. Importantly, inhibition of tumor progression and survival rate was both improved by these focused ultrasound and BCNU combination therapy [35].

In the clinical practice, current standard treatment for newly diagnosed glioma includes maximal safe resection, followed by radiotherapy and chemotherapy, in which the adjuvant temozolomide (TMZ) was used up to six cycles [3, 36]. However, the median survival of standard treatment was still only 4.0 months [37]. The main reason may lie in the poor TMZ delivery efficiency. To overcome this problem, Liu et al. revealed increased TMZ delivery into glioma-bearing mice after being treated with FUS and microbubbles [38]. TMZ degradation time also increased from 1.02 to 1.56 hours on U87 glioma animal models, and also FUS-BBB with TMZ effectively inhibited the tumor growth. In another report, Beccaria et al. used ultrasound to open the BBB, thus significantly enhancing the concentration of both temozolomide (TMZ) and irinotecan (CPT-11) in New Zealand white rabbits [39]. Different with focused ultrasound equipments, in their experiment, sonication was carried out by using the 1.05-MHz planar transducer with injection of microbubbles.

### 1.3. Drug-Loaded Microbubbles for Drug Delivery in Glioma Treatment

In recent years, some advances can be seen in the drug-loaded microbubbles for drug delivery for glioma treatment. Boron neutron capture therapy (BNCT) showed some clinical effect in patients with high-grade gliomas [40]. However, successful treatment of glioma by BNCT requires more efficient boron delivery agents [41, 42]. For a more efficient tumor-targeted delivery of boron, Fan et al. fabricated PEG-b-PMBSH-Loaded MBs, which are formed by boron-containing nanoparticles (PEG-b-PMBSH), coupling with microbubbles, for GL261-bearing mouse glioma model treatment [43]. LA-ICP-MS measurement showed quick uptake of boron in tumor after FUS, with 3-fold increase in the tumor-to-normal-brain ratio. Chemotherapeutic drug-BCNU was encapsulated in the phospholipids of the MB shell by Ting et al., with a loading capacity of 68.01 ± 4.35% and significantly prolonging BCNU' half-life by 5-fold [44]. FUS insonation was applied on tumor-implanted rats after injection with BCNU-MBs, demonstrating excellent tumor progression inhibition, with median survival being significantly prolonged.

Gliomas have been reported to be highly expressed with vascular endothelial growth factor (VEGF) which promotes angiogenesis of tumor. Fan et al. designed VEGF-targeting drug-loaded microbubbles, by conjugating VEGF-A ligand to BCNU-loaded microbubbles (VEGF-BCNU-MBs). It significantly enhanced the glioma-targeted BCNU release and reduced the glioma progression in rat tumor models after the combination of FUS-BBB opening [45]. These microbubbles could open the BBB when exposed to FUS, and it could also extend circulation time of BCNU.

However, the loading capacity of drug-loaded microbubbles is relatively low due to the restricted microbubbles' surface area. To achieve therapeutic effects, the amount injected may need to be increased to levels that should be tested for safety in blood circulation.

### 1.4. BBB Opening and Drug Carriers in Glioma Treatment

Paclitaxel (PTX) is a widely used clinically effective chemotherapy drug. PTX liposomes are developed to deal with its extremely lipophilic nature. Therefore, liposomes significantly increase maximum tolerated dose (MTD) of PTX. However, the delivery of PTX liposomes into gliomas is limited due to the presence of the BBB. Shen et al. showed that the delivery efficiency of PTX liposomes effectively improved when BBB was opened by pulsed FUS sonication. They used FUS parameters with a 10 ms pulse length with microbubbles, improving the therapeutic efficacy of PTX-LIPO in nude mice glioma model [46].

Recently, Li et al. have developed polysorbate 80- (PS-80-) modified PLGA nanoparticles loaded with paclitaxel (PS-80-PTX-NPs, PPNP) for glioma treatment [47]. The size of nanoparticles was 170.5 ± 7.1 nm, with zeta potential of –54.7 ± 0.46 mV. Locally enhanced drug delivery into the brain in vivo was achieved by combining with FUS-BBB opening. The median survival time of U87-luc bearing mice increased to 37 days after being injected with PPNP, compared with the control group (26 days). Immunofluorescence staining of tight junction- (TJ-) related protein (ZO-1) and P-glycoprotein (P-gp) revealed that FUS could disrupt ZO-1 protein and reduce the expression of P-gp. PPNP attached with the ApoE receptor with the help of PS-80 and then activated receptor-mediated endocytosis. Therefore, PPNP and FUS-BBB enhanced the drug delivery and antiglioma efficacy of paclitaxel. However, pharmacokinetics and pharmacodynamics of each kind of drug carriers need to be explored for safety in human use.

### 1.5. FUS-BBB Opening and Magnetic/Ultrasound Focusing System in Glioma Treatment

Focused ultrasound (FUS) can increase the permeability of the BBB, with combination of circulating microbubbles. However, the delivery efficiency of free diffusion of drugs into brain in this passive manner is low, because of the high interstitial fluid pressure (IFP) in gliomas which inhibits convective transportation of drugs. Many strategies have been explored, aiming for antiglioma drug delivery more effectively [48]. For example, an externally applied magnetic field (MT) could achieve targeting of nanoparticles and localized drug delivery in deep-seated gliomas [49, 50]. The combined use of FUS-BBB opening and externally MT could deliver therapeutic particles across the BBB both passively and actively. Chen et al. fabricated Fe_3_O_4_/SPAnH nanoparticles (MNPs) with chemotherapeutic BCNU immobilized on the MNPs [51]. The immobilization ratio was 86.2%, and the size was about 10–20 nm. The magnetic/ultrasound focusing system was developed, aiming for a lower dose of chemotherapeutic and clinical therapy monitor. Firstly, FUS-BBB opening of rodent gliomas increased the passive diffusion of drugs. Subsequently, magnetic field was utilized to enhance localization to gliomas. Quantitative analysis of iron showed that that MT/FUS system increased the particle accumulation by 26-fold, compared with animals only injected with MNPs without MT/FUS. Combination of MT/FUS and BCNU-MNP enhanced the BCNU delivery, efficiently shrinking the tumors.

Liu et al. also developed epirubicin-MNPs in another document. Their data revealed that MNP accumulation in the brain could be detected by T2-weighted MRI imaging [52]. Therapeutic MNPs together with FUS/MT improved deposition of MNPs and controlled the tumor progression in C6-bearing rat glioma models.

Superparamagnetic iron oxide (SPIO) nanoparticles have been approved as clinical MRI diagnosis contrast drug. Fan et al. designed an SPIO-labeled phospholipid-based microbubbles, with doxorubicin (DOX) incorporated in it [53]. This DOX-SPIO-MBs showed uniform size distributions of 1.04 ± 0.01 *μ*m, with R2 relaxivity of 107.3 mM^–1^s^–1^. Magnetic field (MT) was applied after the FUS-BBB opening. SPIO concentration in the tumor was greatly enhanced by 22.4%. DOX-SPIO-MBs could be insonated by FUS, as well as by both MRI and US imaging, to open the BBB, allowing MT to enhance glioma-targeted drug delivery. How to scale up the setup for human applications will be meaningful but challenging.

### 1.6. Image-Guided FUS-BBB Opening and Nanoparticle Delivery in Glioma Treatment

Nanoparticles for both diagnosis and therapy are potential for effective glioma treatment. Diaz et al. fabricated a novel GNPs functionalized with EGFR antibody. Nanoparticles were delivered across the BBB by MRI-guided FUS, enabling spectral mapping of nanoparticles for in vivo tumor tracking [54]. Recently, Zhang et al. developed nanoparticles based on ^99m^Tc-labeled ultrasmall Cu_2–*x*_Se. This nanoparticle enabled dual modal photoacoustic (PA) imaging and SPECT imaging at the same time. Therefore, noninvasive monitoring of the opening of FUS-BBB and the recovery status of BBB in vivo were demonstrated in this research [55].

Magnetic resonance imaging (MRI) has been applied for years to monitor in vivo BBB disruption. To monitor BBB disruptions and of individual vessels, the limited spatial resolution of MRI is not the best choice. Instead of MRI, two-photon microscopy was utilized on rats with a craniotomy by Cho et al. [56]. In this study, various acoustic peak negative pressures were employed for in vivo visualization of vasculature responses. Acoustic pressure could control disruption type and size of the vessel in FUS-BBB opening.

**Figure 2:**
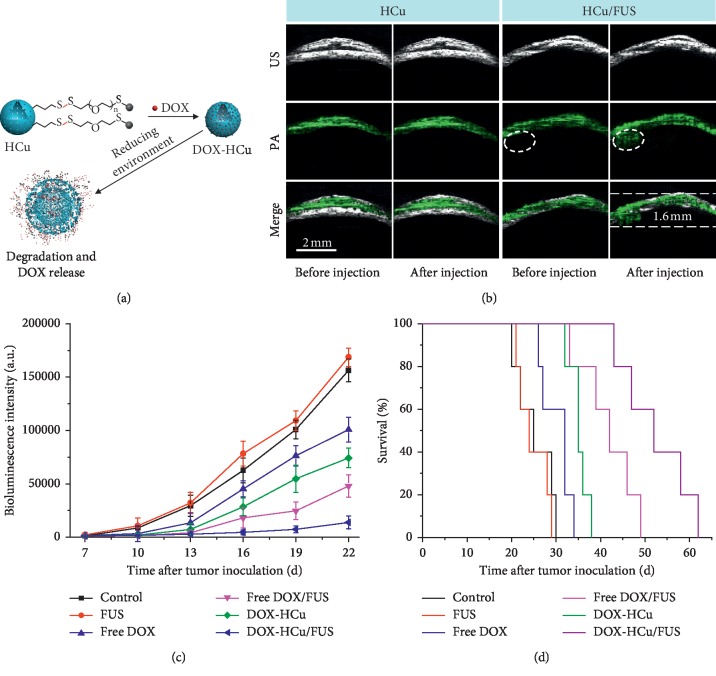
(a) Schematic illustration of DOX-HCu; (b) PA images in the tumor; (c) bioluminescent signal intensity in U87-Luc glioma model; (d) survival curves of U87-bearing mice (reproduced from Reference [58], an open-access article by the authors and published by WileyVCH Verlag GmbH & Co. KGaA, Weinheim).

Photoacoustic imaging demonstrated the potential to overcome the limitations of optical imaging, allowing visualization of deep-seated glioma [57]. Recently, Wu et al. constructed novel theranostic nanosystems for glioma treatment under PA imaging [58]. In this study, Cu_2–*x*_Se-PEG-SH was grafted onto hollow mesoporous organosilica nanoparticles (HMONs), enabling excellent PA images in the orthotopic U87-Luc glioma beneath the mouse skull. Doxorubicin (DOX) was loaded into the hollow interior of nanoparticles, designated as DOX-HCu. Combined with FUS-BBB opening, DOX-HCu demonstrated great inhibition of tumor progression. The median survival time of U87-bearing mice treated with DOX-HCu and FUS was fifty-two days, much longer than mice treated with DOX-HCu (thirty-five days), as shown in Figure 2. However, the safety and efficacy of theranostics still need to be intensively tested for clinical use. And in vivo stability, degradation, and clearance of nanoparticles are also important aspects for clinical translation.

### 1.7. FUS-BBB Opening and Gene Therapy in Glioma Treatment

**Figure 3:**
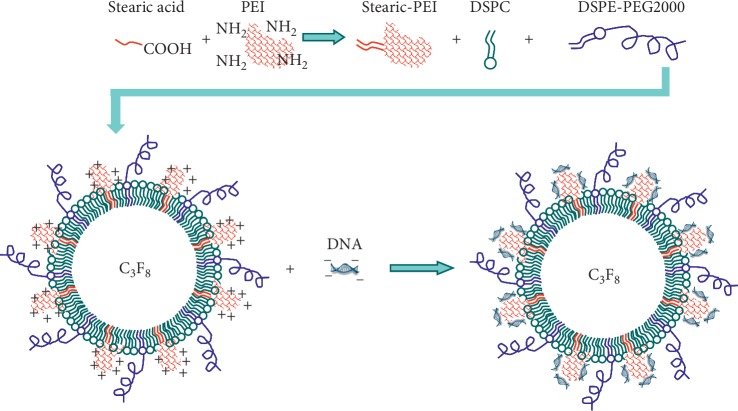
Schematic illustration of DNA-loading microbubbles (reproduced from Reference [60], an open-access article published by PLOS).

Gene therapy promises for effective treatment of glioma. Mead et al. realized systemically administered DNA-bearing BPN (DNA-BPN) delivery across the BBB. Localized and sustained transgene expression was obtained in the rat brain [59]. In this study, PEGylated DNA-BPN based on polyethylenimine (PEI) was formulated. FUS-BBB opening enhanced the DNA-BPN delivery across the BBB, showing astrocytes and neurons transfected in the brain. Jin et al. designed a novel DNA-loading microbubble, showing potential as a gene delivery carrier, as shown in Figure 3 [60].

Targeted delivery of “suicide” genes into gliomas is believed to augment sensitivity to select prodrugs. Locally delivered gene therapy for glioma is important for patients who do not respond to standard chemotherapy [61]. Recently, Pan et al. demonstrated noninvasive and local suicide gene delivery can be achieved. In their study, FUS was used for BBB opening, and herpes simplex virus thymidine kinase gene was delivered to glioma. After exposure to GCV, stronger in vivo antiglioma efficacy and longer survival time were observed in glioma-bearing rats [62]. There are still many works need to be done for clinical translation of gene therapy in glioma treatment.

### 1.8. FUS-BBB Opening and Immunotherapy in Glioma Treatment

Glioma frequently undergoes relapses in clinics, because of the resistance to chemotherapy and radiation therapy. Immunotherapy may represent a promising approach, aiming to eliminate residual glioma cells [63]. Usually, immunological tolerance of brain tumors may partially overcome host immune defenses, which limits primary immune responses [64]. IL-12 had been reported to stimulate an antitumor immune response, exerting anti-angiogenic and antiglioma effects. Fifteen patients with malignant gliomas participated to test the safety and clinical response of recombinant human interleukin 12 (rhIL-12) [65]. Chen et al. reported that FUS-BBB opening enhanced the immune-modulating agent in C-6 glioma bearing rats [66]. They found that combination of FUS-BBB increased IL-12 significantly, leading to increase of CTL and CTL/Treg ratio, tumor progression suppression, and animal survival prolonging.

FUS-BBB opening is promising for the delivery of antibody-based anticancer therapy. Bevacizumab, a VEGF antibody, was approved by the FDA for recurrent glioma treatment. Liu et al. reported that FUS-enhanced bevacizumab delivery significantly enhanced its penetration into glioma and retarded the tumor progression with a significantly increased median survival [67]. Kinoshita reported that Herceptin, a monoclonal antibody against HER2, can also be delivered into the mouse brain through the BBB by using MRI-guided FUS-BBB opening technique [68]. Before these techniques were widely used, biodistribution of immunotherapy and safety concerns need to be understood.

### 1.9. Clinical Trials

Alexandre et al. carried out a phase 1/2a trial to test the safety and efficacy of the implantable SonoCloud device, for BBB opening by pulse US repeatedly [69]. The BBB of patient was opened monthly using pulsed US and microbubbles. Presently, MRI-guided FUS has already been used in several clinical trials for the treatment of cerebral tumors [70]. Five patients with glioma were enrolled in the clinical trial (NCT0234399), and the BBB was safely and successfully opened as shown by gadolinium enhancement by MRI [71]. And, transient FUS-BBB opening with chemotherapy was safe and feasible.

## 2. Conclusions and Perspectives

Effective delivery of various diagnostic or therapeutic agents across the blood-brain-barrier (BBB) remains a major challenge in the treatment of glioma. Focused ultrasound (FUS) combined with microbubbles is potential for noninvasively and reversibly disrupting the BBB. For technical optimization and further clinical application of this technology, intensive studies are required to focus on the thorough understanding of exact mechanisms involved in the FUS-mediated disruption of the BBB.

## References

[1] Wen P. Y., Kesari S. (2008). Malignant gliomas in adults. *New England Journal of Medicine*.

[2] Omuro A., DeAngelis L. M. (2013). Glioblastoma and other malignant gliomas. *Jama*.

[3] Stupp R., Mason W. P., van den Bent M. J. (2005). Radiotherapy plus concomitant and adjuvant temozolomide for glioblastoma. *New England Journal of Medicine*.

[4] Abbott N. J., Rönnbäck L., Hansson E. (2006). Astrocyte-endothelial interactions at the blood-brain barrier. *Nature Reviews Neuroscience*.

[5] Luissint A.-C., Artus C., Glacial F., Ganeshamoorthy K., Couraud P.-O. (2012). Tight junctions at the blood brain barrier: physiological architecture and disease-associated dysregulation. *Fluids and Barriers of the CNS*.

[6] Obermeier B., Daneman R., Ransohoff R. M. (2013). Development, maintenance and disruption of the blood-brain barrier. *Nature Medicine*.

[7] Liu H.-L., Fan C.-H., Ting C.-Y., Yeh C.-K. (2014). Combining microbubbles and ultrasound for drug delivery to brain tumors: current progress and overview. *Theranostics*.

[8] Pardridge W. M. (2002). Drug and gene delivery to the brain. *Neuron*.

[9] Ewing J. R., Brown S. L., Lu M. (2006). Model selection in magnetic resonance imaging measurements of vascular permeability: gadomer in a 9L model of rat cerebral tumor. *Journal of Cerebral Blood Flow & Metabolism*.

[10] Brem H., Piantadosi S., Burger P. C. (1995). Placebo-controlled trial of safety and efficacy of intraoperative controlled delivery by biodegradable polymers of chemotherapy for recurrent gliomas. *The Lancet*.

[11] Souweidane M. M., Kramer K., Pandit-Taskar N. (2018). Convection-enhanced delivery for diffuse intrinsic pontine glioma: a single-centre, dose-escalation, phase 1 trial. *The Lancet Oncology*.

[12] Allard E., Passirani C., Benoit J.-P. (2009). Convection-enhanced delivery of nanocarriers for the treatment of brain tumors. *Biomaterials*.

[13] Jolesz F. A., Hynynen K., McDannold N., Tempany C. (2005). MR imaging-controlled focused ultrasound ablation: a noninvasive image-guided surgery. *Magnetic Resonance Imaging Clinics of North America*.

[14] Hynynen K., McDannold N., Vykhodtseva N. (2006). Focal disruption of the blood-brain barrier due to 260-kHz ultrasound bursts: a method for molecular imaging and targeted drug delivery. *Journal of Neurosurgery*.

[15] Sheikov N., McDannold N., Sharma S., Hynynen K. (2008). Effect of focused ultrasound applied with an ultrasound contrast agent on the tight junctional integrity of the brain microvascular endothelium. *Ultrasound in Medicine & Biology*.

[16] Kang S. T., Yeh C. K. (2012). Ultrasound microbubble contrast agents for diagnostic and therapeutic applications: current status and future design. *Chang Gung Medical Journal*.

[17] Shang X., Wang P., Liu Y., Zhang Z., Xue Y. (2011). Mechanism of low-frequency ultrasound in opening blood-tumor barrier by tight junction. *Journal of Molecular Neuroscience*.

[18] Hynynen K., McDannold N., Vykhodtseva N., Jolesz F. A. (2001). Noninvasive MR imaging-guided focal opening of the blood-brain barrier in rabbits. *Radiology*.

[19] McDannold N., Vykhodtseva N., Hynynen K. (2007). Use of ultrasound pulses combined with definity for targeted blood-brain barrier disruption: a feasibility study. *Ultrasound in Medicine & Biology*.

[20] Hernot S., Klibanov A. L. (2008). Microbubbles in ultrasound-triggered drug and gene delivery. *Advanced Drug Delivery Reviews*.

[21] Bing C. C., Hong Y., Hernandez C. (2018). Characterization of different bubble formulations for blood-brain barrier opening using a focused ultrasound system with acoustic feedback control. *Scientific Reports*.

[22] Choi J. J., Selert K., Vlachos F., Wong A., Konofagou E. E. (2011). Noninvasive and localized neuronal delivery using short ultrasonic pulses and microbubbles. *Proceedings of the National Academy of Sciences*.

[23] Chen H., Konofagou E. E. (2014). The size of blood-brain barrier opening induced by focused ultrasound is dictated by the acoustic pressure. *Journal of Cerebral Blood Flow & Metabolism*.

[24] Chopra R., Vykhodtseva N., Hynynen K. (2010). Influence of exposure time and pressure amplitude on blood-brain-barrier opening using transcranial ultrasound exposures. *ACS Chemical Neuroscience*.

[25] Mcdannold N., Vykhodtseva N., Hynynen K. (2008). Effects of acoustic parameters and ultrasound contrast agent dose on focused-ultrasound induced blood-brain barrier disruption. *Ultrasound in Medicine & Biology*.

[26] Wu S. K., Chu P. C., Chai W. Y. (2017). Characterization of different microbubbles in assisting focused ultrasound-induced blood-brain barrier opening. *Scientific Reports*.

[27] Marquet F., Tung Y. S., Teichert T., Ferrera V. P., Konofagou E. E. (2011). Noninvasive, transient and selective blood-brain barrier opening in non-human primates in vivo. *PLoS One*.

[28] Samiotaki G., Konofagou E. E. (2013). Dependence of the reversibility of focused- ultrasound-induced blood-brain barrier opening on pressure and pulse length in vivo. *IEEE Transactions on Ultrasonics, Ferroelectrics, and Frequency Control*.

[29] McMahon D., Hynynen K. (2017). Acute inflammatory response following increased blood-brain barrier permeability induced by focused ultrasound is dependent on microbubble dose. *Theranostics*.

[30] Tung Y.-S., Vlachos F., Feshitan J. A., Borden M. A., Konofagou E. E. (2011). The mechanism of interaction between focused ultrasound and microbubbles in blood-brain barrier opening in mice. *The Journal of the Acoustical Society of America*.

[31] McMahon D., Poon C., Hynynen K. (2019). Evaluating the safety profile of focused ultrasound and microbubble-mediated treatments to increase blood-brain barrier permeability. *Expert Opinion on Drug Delivery*.

[32] Meng Y., Pople C. B., Lea-Banks H. (2019). Safety and efficacy of focused ultrasound induced blood-brain barrier opening, an integrative review of animal and human studies. *Journal of Controlled Release*.

[33] Maimbourg G., Houdouin A., Santin M., Lehericy S., Tanter M., Aubry J.-F. (2018). Inside/outside the brain binary cavitation localization based on the lowpass filter effect of the skull on the harmonic content: a proof of concept study. *Physics in Medicine & Biology*.

[34] Treat L. H., McDannold N., Vykhodtseva N., Zhang Y., Tam K., Hynynen K. (2007). Targeted delivery of doxorubicin to the rat brain at therapeutic levels using MRI-guided focused ultrasound. *International Journal of Cancer*.

[35] Liu H.-L., Hua M.-Y., Chen P.-Y. (2010). Blood-brain barrier disruption with focused ultrasound enhances delivery of chemotherapeutic drugs for glioblastoma treatment. *Radiology*.

[36] Stupp R., Hegi M. E., Mason W. P. (2009). Effects of radiotherapy with concomitant and adjuvant temozolomide versus radiotherapy alone on survival in glioblastoma in a randomised phase III study: 5-year analysis of the EORTC-NCIC trial. *The Lancet Oncology*.

[37] Wasserfallen J.-B., Ostermann S., Leyvraz S., Stupp R. (2005). Cost of temozolomide therapy and global care for recurrent malignant gliomas followed until death. *Neuro-Oncology*.

[38] Liu H. L., Huang C. Y., Chen J. Y., Wang H. Y., Chen P. Y., Wei K. C. (2014). Pharmacodynamic and therapeutic investigation of focused ultrasound-induced blood-brain barrier opening for enhanced temozolomide delivery in glioma treatment. *PLoS One*.

[39] Beccaria K., Canney M., Goldwirt L. (2016). Ultrasound-induced opening of the blood-brain barrier to enhance temozolomide and irinotecan delivery: an experimental study in rabbits. *Journal of Neurosurgery*.

[40] Barth R. F., Coderre J. A., Vicente M. G., Blue T. E. (2005). Boron neutron capture therapy of cancer: current status and future prospects. *Clinical Cancer Research*.

[41] Maruyama K., Ishida O., Kasaoka S. (2004). Intracellular targeting of sodium mercaptoundecahydrododecaborate (BSH) to solid tumors by transferrin-PEG liposomes, for boron neutron-capture therapy (BNCT). *Journal of Controlled Release*.

[42] Meo C. D., Panza L., Capitani D. (2007). Hyaluronan as carrier of carboranes for tumor targeting in boron neutron capture therapy. *Biomacromolecules*.

[43] Fan C.-H., Wang T.-W., Hsieh Y.-K. (2019). Enhancing boron uptake in brain glioma by a boron-polymer/microbubble complex with focused ultrasound. *ACS Applied Materials & Interfaces*.

[44] Ting C.-Y., Fan C.-H., Liu H.-L. (2012). Concurrent blood-brain barrier opening and local drug delivery using drug-carrying microbubbles and focused ultrasound for brain glioma treatment. *Biomaterials*.

[45] Fan C.-H., Ting C.-Y., Liu H.-L. (2013). Antiangiogenic-targeting drug-loaded microbubbles combined with focused ultrasound for glioma treatment. *Biomaterials*.

[46] Shen Y., Pi Z., Yan F. (2017). Enhanced delivery of paclitaxel liposomes using focused ultrasound with microbubbles for treating nude mice bearing intracranial glioblastoma xenografts. *International Journal of Nanomedicine*.

[47] Li Y., Wu M., Zhang N. (2018). Mechanisms of enhanced antiglioma efficacy of polysorbate 80-modified paclitaxel-loaded PLGA nanoparticles by focused ultrasound. *Journal of Cellular and Molecular Medicine*.

[48] Groothuis D. R. (2000). The blood-brain and blood-tumor barriers: a review of strategies for increasing drug delivery. *Neuro-Oncology*.

[49] Alexiou C., Arnold W., Klein R. J. (2000). Locoregional cancer treatment with magnetic drug targeting. *Cancer Research*.

[50] Mody V. V., Cox A., Shah S., Singh A., Bevins W., Parihar H. (2014). Magnetic nanoparticle drug delivery systems for targeting tumor. *Applied Nanoscience*.

[51] Chen P.-Y., Liu H.-L., Hua M.-Y. (2010). Novel magnetic/ultrasound focusing system enhances nanoparticle drug delivery for glioma treatment. *Neuro-Oncology*.

[52] Liu H.-L., Hua M.-Y., Yang H.-W. (2010). Magnetic resonance monitoring of focused ultrasound/magnetic nanoparticle targeting delivery of therapeutic agents to the brain. *Proceedings of the National Academy of Sciences*.

[53] Fan C.-H., Ting C.-Y., Lin H.-J. (2013). SPIO-conjugated, doxorubicin-loaded microbubbles for concurrent MRI and focused-ultrasound enhanced brain-tumor drug delivery. *Biomaterials*.

[54] Diaz R. J., McVeigh P. Z., O’Reilly M. A. (2014). Focused ultrasound delivery of Raman nanoparticles across the blood-brain barrier: potential for targeting experimental brain tumors. *Nanomedicine: Nanotechnology, Biology, and Medicine*.

[55] Zhang H., Wang T., Qiu W. (2018). Monitoring the opening and recovery of the blood-brain barrier with noninvasive molecular imaging by biodegradable ultrasmall Cu_2-*x*_Se nanoparticles. *Nano Letters*.

[56] Cho E. E., Drazic J., Ganguly M., Stefanovic B., Hynynen K. (2011). Two-photon fluorescence microscopy study of cerebrovascular dynamics in ultrasound-induced blood-brain barrier opening. *Journal of Cerebral Blood Flow & Metabolism*.

[57] Valluru K. S., Wilson K. E., Willmann J. K. (2016). Photoacoustic imaging in oncology: translational preclinical and early clinical experience. *Radiology*.

[58] Wu M., Chen W., Chen Y. (2018). Focused ultrasound-augmented delivery of biodegradable multifunctional nanoplatforms for imaging-guided brain tumor treatment. *Advanced Science*.

[59] Mead B. P., Mastorakos P., Suk J. S., Klibanov A. L., Hanes J., Price R. J. (2016). Targeted gene transfer to the brain via the delivery of brain-penetrating DNA nanoparticles with focused ultrasound. *Journal of Controlled Release*.

[60] Jin Q., Wang Z., Yan F. (2013). A novel cationic microbubble coated with stearic acid-modified polyethylenimine to enhance DNA loading and gene delivery by ultrasound. *PLoS One*.

[61] Westphal M., Ylä-Herttuala S., Martin J. (2013). Adenovirus-mediated gene therapy with sitimagene ceradenovec followed by intravenous ganciclovir for patients with operable high-grade glioma (ASPECT): a randomised, open-label, phase 3 trial. *The Lancet Oncology*.

[62] Pan M., Zhang Y., Deng Z., Yan F., Hong G. (2018). Noninvasive and local delivery of adenoviral-mediated herpes simplex virus thymidine kinase to treat glioma through focused ultrasound-induced blood-brain barrier opening in rats. *Journal of Biomedical Nanotechnology*.

[63] Schumacher T., Bunse L., Pusch S. (2014). A vaccine targeting mutant IDH1 induces antitumour immunity. *Nature*.

[64] Okada H. (2009). Brain tumor immunotherapy with type-1 polarizing strategies. *Annals of the New York Academy of Sciences*.

[65] Kikuchi T., Akasaki Y., Abe T. (2004). Vaccination of glioma patients with fusions of dendritic and glioma cells and recombinant human interleukin 12. *Journal of Immunotherapy*.

[66] Chen P.-Y., Hsieh H.-Y., Huang C.-Y., Lin C.-Y., Wei K.-C., Liu H.-L. (2015). Focused ultrasound-induced blood-brain barrier opening to enhance interleukin-12 delivery for brain tumor immunotherapy: a preclinical feasibility study. *Journal of Translational Medicine*.

[67] Liu H.-L., Hsu P.-H., Lin C.-Y. (2016). Focused ultrasound enhances central nervous system delivery of Bevacizumab for malignant glioma treatment. *Radiology*.

[68] Kinoshita M., McDannold N., Jolesz F. A., Hynynen K. (2006). Noninvasive localized delivery of Herceptin to the mouse brain by MRI-guided focused ultrasound-induced blood-brain barrier disruption. *Proceedings of the National Academy of Sciences*.

[69] Carpentier A., Canney M., Vignot A. (2016). Clinical trial of blood-brain barrier disruption by pulsed ultrasound. *Science Translational Medicine*.

[70] Toccaceli G., Barbagallo G., Peschillo S. (2019). Low-intensity focused ultrasound for the treatment of brain diseases: safety and feasibility. *Theranostics*.

[71] Mainprize T., Lipsman N., Huang Y. (2019). Blood-brain barrier opening in primary brain tumors with non-invasive MR-guided focused ultrasound: a clinical safety and feasibility study. *Scientific Reports*.

